# Supplementary material for: Loss of colonic fidelity enables multilineage plasticity and metastasis
Source: Nature. 2025 Jun 4;644(8076):547–56. doi: 10.1038/s41586-025-09125-5 (PMC12350155; doi:10.1038/s41586-025-09125-5)
Supplement: Supplementary file 1 — Supplementary Information 1–3. [file 41586_2025_9125_MOESM1_ESM.pdf]

nature portfolio

https://doi.org/10.1038/s41586-025-09125-5

Supplementary information

Loss of colonic fidelity enables multilineage plasticity and metastasis

In the format provided by the
authors and unedited

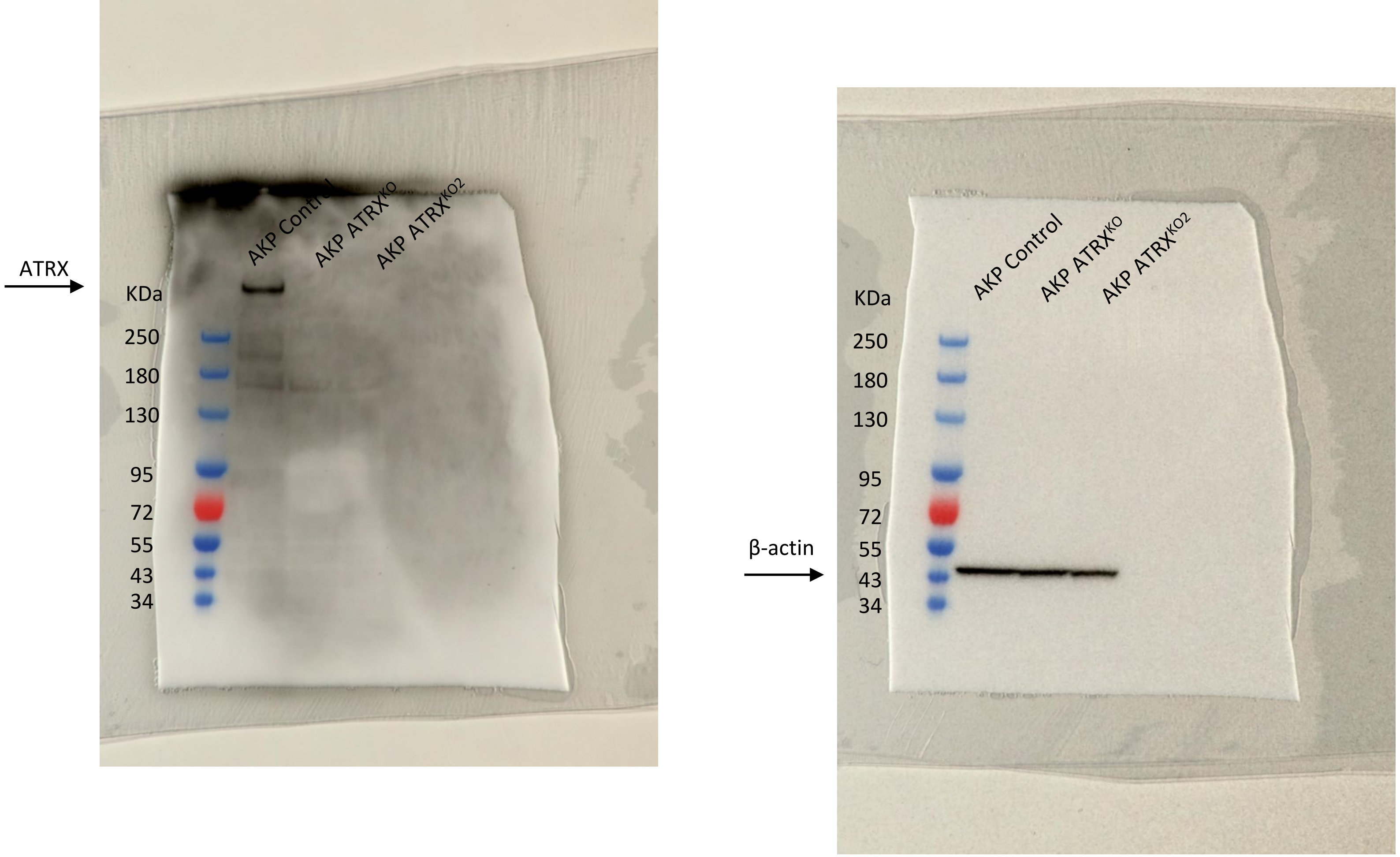

Supplementary Information 1. Uncropped western-blot related to Extended Data Fig. 1h.

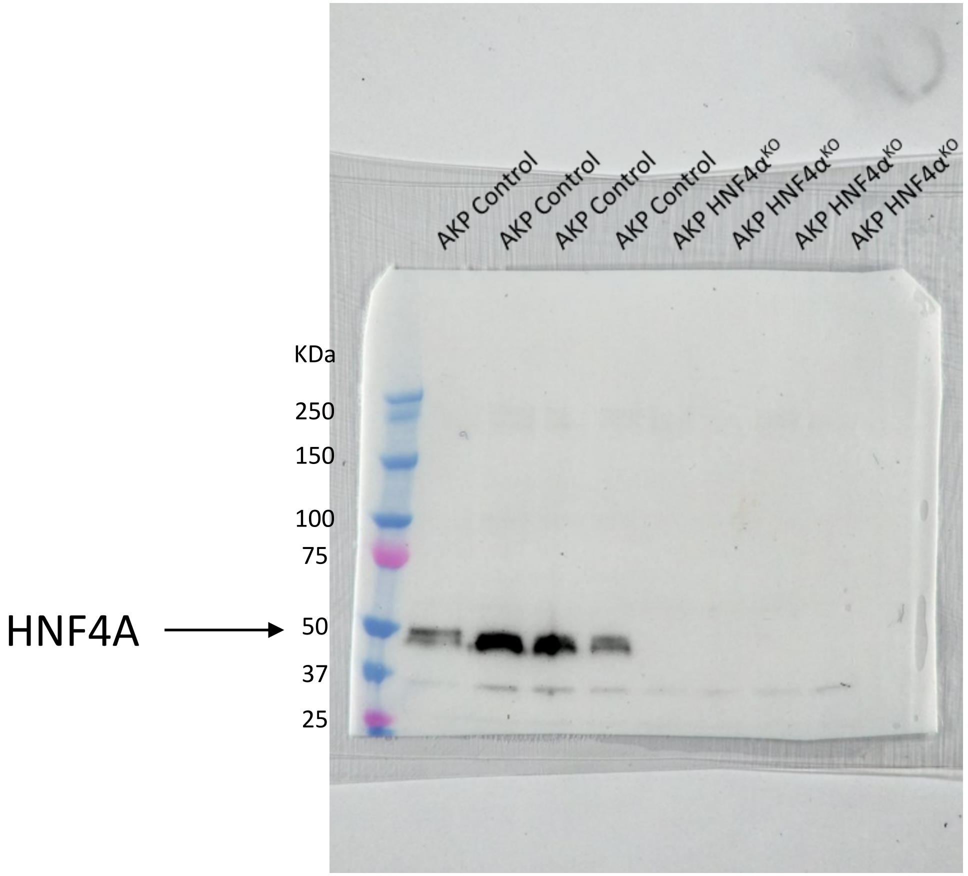

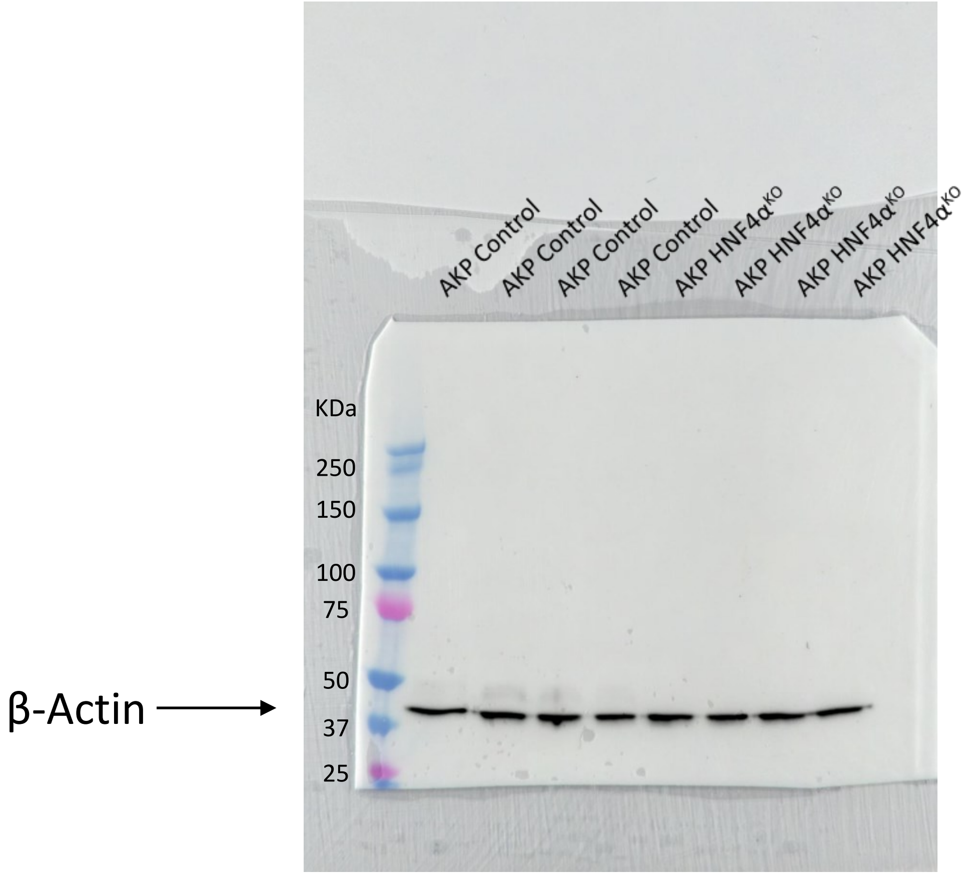

Supplementary Information 2. Uncropped western-blot related to Extended Data Fig. 9i

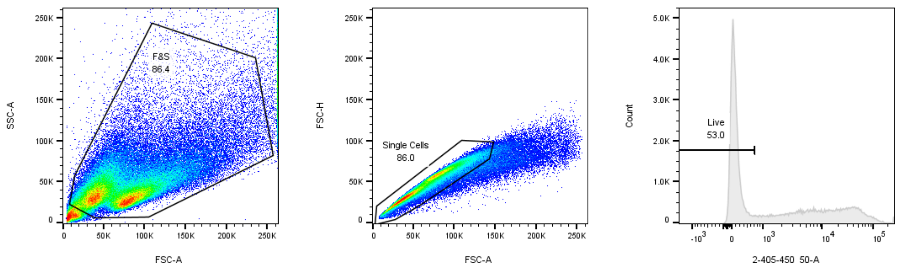

Supplementary Information 3. Representative example of gating strategy used in study
