# Supplementary material for: Loss of colonic fidelity enables multilineage plasticity and metastasis
Source: Nature. 2025 Jun 4;644(8076):547–56. doi: 10.1038/s41586-025-09125-5 (PMC12350155; doi:10.1038/s41586-025-09125-5)
Supplement: Supplementary file 2 — Reporting Summary [file 41586_2025_9125_MOESM2_ESM.pdf]

nature portfolio

Corresponding author(s): Kevin Myant

Last updated by author(s): Apr 29, 2025

Reporting Summary

Nature Portfolio wishes to improve the reproducibility of the work that we publish. This form provides structure for consistency and transparency in reporting. For further information on Nature Portfolio policies, see our Editorial Policies and the Editorial Policy Checklist.

Statistics

For all statistical analyses, confirm that the following items are present in the figure legend, table legend, main text, or Methods section.

n/a	Confirmed	
☐	☒	The exact sample size (*n*) for each experimental group/condition, given as a discrete number and unit of measurement
☐	☒	A statement on whether measurements were taken from distinct samples or whether the same sample was measured repeatedly
☐	☒	The statistical test(s) used AND whether they are one- or two-sided<br>*Only common tests should be described solely by name; describe more complex techniques in the Methods section.*
☒	☐	A description of all covariates tested
☐	☒	A description of any assumptions or corrections, such as tests of normality and adjustment for multiple comparisons
☐	☒	A full description of the statistical parameters including central tendency (e.g. means) or other basic estimates (e.g. regression coefficient) AND variation (e.g. standard deviation) or associated estimates of uncertainty (e.g. confidence intervals)
☐	☒	For null hypothesis testing, the test statistic (e.g. *F*, *t*, *r*) with confidence intervals, effect sizes, degrees of freedom and *P* value noted<br>*Give P values as exact values whenever suitable.*
☒	☐	For Bayesian analysis, information on the choice of priors and Markov chain Monte Carlo settings
☒	☐	For hierarchical and complex designs, identification of the appropriate level for tests and full reporting of outcomes
☐	☒	Estimates of effect sizes (e.g. Cohen's *d*, Pearson's *r*), indicating how they were calculated

Our web collection on statistics for biologists contains articles on many of the points above.

Software and code

Policy information about availability of computer code

Data collection	RNAseq, ATAC-seq, CUT&RUN, 10x scRNAseq: NovaseqS1 Illumina sequencing, NextSeq 2000 platform.<br>qPCR: BioRad CFX Connect<br>Histology: Hamamatsu Nanozoomer XR<br>Flow cytometry and cell sorting (FACS): BD FACS ARIA II/BD LSR-Fortessa X-20
Data analysis	RNAseq analysis - RaNASeq pipeline, GSEA, TissueEnrich. ATAC-seq - DESeq2, monaLisa R package, IGV browser. CUT&RUN - MACS2, Diffbind, BEDTools. Histology - NDP.view2 U12388-01, QuPath 0.2.3. Graphs and statistics - Microsoft Office Excel 365, GraphPad Prism 9.0. 10x scRNAseq - Cell Ranger 7.2.0, R package Seurat (v5), TissueEnrich. FACS - FlowJo v10.8.0.

For manuscripts utilizing custom algorithms or software that are central to the research but not yet described in published literature, software must be made available to editors and reviewers. We strongly encourage code deposition in a community repository (e.g. GitHub). See the Nature Portfolio guidelines for submitting code & software for further information.

Data

Policy information about availability of data

All manuscripts must include a data availability statement. This statement should provide the following information, where applicable:

- Accession codes, unique identifiers, or web links for publicly available datasets
- A description of any restrictions on data availability
- For clinical datasets or third party data, please ensure that the statement adheres to our policy

The CUT&RUN, ATAC-seq, scRNA seq and RNAseq data generated in this study are deposited in the Genome Sequence Archive (https://ngdc.cncb.ac.cn/gsa/) website. The accession numbers for the data are: CRA024850 (H3K27ac AKP vs AKP ATRX); CRA024849 (ATACseq AKP vs AKP ATRX); CRA024816 (scRNAseq AKP vs AKP ATRX; CRA024804 (RNAseq AKP vs AKP HNF4A); CRA024763 (RNAseq AKP vs AKP ATRX).

Extended data Figure 4p available from Human Protein Atlas proteinatlas.org

All data are provided within the article and Supplementary Information.

Research involving human participants, their data, or biological material

Policy information about studies with human participants or human data. See also policy information about sex, gender (identity/presentation), and sexual orientation and race, ethnicity and racism.

Reporting on sex and gender	Tissues were obtained from both males and females.
Reporting on race, ethnicity, or other socially relevant groupings	n/a
Population characteristics	This study did not involve human research participants, but utilised human derived biospecimens to generate primary organoids or for FACS analysis. Patient samples were collected from daily surgery and researchers were blinded to the identification of donors. Available patients information are reported in supplementary table and method section.
Recruitment	Patients were recruited as having primary colorectal tumours in order to derive tumour material to investigate mechanisms important for this disease. No biases were present that might likely impact results.
Ethics oversight	Ethical approval for human CRC organoid derivation was carried out under NHS Lothian Ethical Approval Scottish Colorectal Cancer Genetic Susceptibility Study 3 (SOCCS3) (REC reference: 11/SS/0109). All patients provided fully informed consent for use of their tissues.<br><br>In-house TMA: patient tissue access was authorized by the NHS Greater Glasgow and Clyde Biorepository under their NHS Research Ethics Committee approval with ethical approval granted in biorepository application #845, West of Scotland Ethics 22/WS/0207 in accordance with recognized ethical guidelines as described in the Declaration of Helsinki.<br><br>17 patients undergoing synchronous resection of primary colorectal cancer and colorectal cancer liver metastases with curative intent between April 2002 and June 2010 at Glasgow Royal Infirmary (UK) were analysed. Patient tissue access was authorized by the NHS Greater Glasgow and Clyde Biorepository under their NHS Research Ethics Committee approval with ethical approval granted in biorepository application #357, West of Scotland Ethics 22/WS/0207 in accordance with recognized ethical guidelines as described in the Declaration of Helsinki.

Note that full information on the approval of the study protocol must also be provided in the manuscript.

Field-specific reporting

Please select the one below that is the best fit for your research. If you are not sure, read the appropriate sections before making your selection.

☒ Life sciences ☐ Behavioural & social sciences ☐ Ecological, evolutionary & environmental sciences

For a reference copy of the document with all sections, see nature.com/documents/nr-reporting-summary-flat.pdf

Life sciences study design

All studies must disclose on these points even when the disclosure is negative.

Sample size	Sample sizes for each experiment are outlined in the figure legends.<br><br>For all animal experiments, n > 5 mice were used for each experimental cohort. Power analyses were carried out prior to experiments being carried out to determine the minimum number of animals required for each experiment. These analyses were informed by previous and / or preliminary experiments (for example Gudino et al Nat Comms, 2021).

	For organoid experiments, all are derived from n = 3 or n > 3 independent experiments unless otherwise stated. Sample sizes were not statistically predetermined and were based on the results of previous published experiments with these models (for example Gudino et al Nat Comms, 2021).<br><br>For RNAseq, ATAC-seq and CUT&RUN experiments, all are derived from n = 3 independent samples. Sample sizes were not statistically predetermined and were based on the results of previous experiments with these models (for example Gudino et al Nat Comms, 2021).<br><br>For scRNAseq experiment, all are derives from n = 2 independent samples per condition.
Data exclusions	No data were excluded from analysis.
Replication	Experiments (mouse, RNAseq, ATAC-seq, CUT&RUN, RT-qPCR, organoids) were replicated at least 3 times using the same experimental approach or using multiple biologically independent replicates. scRNAseq : 2 technical replicates per condition. All replication attempts were successful.
Randomization	C57/B6J or CD1 nude mice of ages 6-12 weeks were randomly grouped for transplantation experiments. All mice received the same number of cells of different genotype / phenotype. Experimental groups were determined by genotype of injected cells (for example AKP vs AKP AtrxKO).
Blinding	Investigators were blinded to the genotype of tumours when monitoring for clinical signs, when carrying out histological analysis and during data collection. IHC analysis of tumour histology was carried out using QuPath software with the investigator blinded to tumour genotype. For the in vitro experiments data collection and analysis were not performed blinded to the condition of the experiments.

Reporting for specific materials, systems and methods

We require information from authors about some types of materials, experimental systems and methods used in many studies. Here, indicate whether each material, system or method listed is relevant to your study. If you are not sure if a list item applies to your research, read the appropriate section before selecting a response.

Materials & experimental systems

n/a	Involved in the study
☐	☒ Antibodies
☐	☒ Eukaryotic cell lines
☒	☐ Palaeontology and archaeology
☐	☒ Animals and other organisms
☒	☐ Clinical data
☒	☐ Dual use research of concern
☒	☐ Plants

Methods

n/a	Involved in the study
☒	☐ ChIP-seq
☐	☒ Flow cytometry
☒	☐ MRI-based neuroimaging

Antibodies

Antibodies used	Only commercial antibody have been used.<br><br>KRT5 (Rabbit; Abcam 52635 (EP1601Y); 1:200)<br>KRT5 (Chicken; BioLegend 905903; 1:200)<br>LY6D (rabbit; Atlas HPA024755; 1:200)<br>TWIST (mouse; Santa Cruz 81417, 1:200)<br>EpCAM (rabbit; Abcam 71916; 1:200)<br>HNF4α (rabbit; CST 3113 (C11F12), 1:500)<br>ATRX (mouse; Sigma MABE1798, 39F; 1:500)<br>β-catenin (mouse; BD 610154; 1:50)<br>E-cadherin (rabbit; CST 3195, 1:200)<br>CDX2 (mouse; Atlas AMAb 91828, 1:1000)<br>EpCAM-APC (BioLegend 118213; 1:200)<br>LY6D-PE (BioLegend 138603; 1:200)<br>LY6D-APC (Miltenyi 130-115-313; 1:50)<br>ITGA5-PE (BioLegend 103805; 1:200)<br>EpCAM-APC (BioLegend 324207; 1:50)<br>LY6D-FITC (Cusabio Biotech CSB-PA613492LC01HU; 1:50)<br>anti-rabbit-594 (Invitrogen A21207; 1:400)<br>anti-chicken-488 (Invitrogen A78948; 1:400)<br>anti-streptavidin-647 (Invitrogen S32357; 1:400)<br>anti-rabbit-488 (Abcam 150073; 1:400)<br>anti-b-actin (CST 4970, (13E5); 1:5000)<br>anti-rabbit IgG HRP-linked, (CST 7074; 1:5000)<br>anti-mouse IgG HRP-linked, (CST 7076; 1:5000)<br>anti-histone H3, acetyl K27 (rabbit; Abcam 1/250)

Validation	KRT5 (Rabbit; Abcam 52635; 1:200) - Validated by Protein Atlas and by positive signal in skin and negative signal in colon<br>KRT5 (Chicken; BioLegend 905903; 1:200) - Validated by positive signal in skin and negative signal in colon<br>LY6D (rabbit; Atlas HPA024755; 1:200) - Validated by Protein Atlas and by positive signal in skin and negative signal in colon<br>TWIST (mouse; Santa Cruz 81417, 1:200) - Validated by positive signal in cells known to express it<br>EpCAM (rabbit; Abcam 71916; 1:200) - Validated by positive signal in cells known to express it<br>HNF4α (rabbit; CST 3113; 1:500) - Validated by positive signal in cells known to express it<br>ATRX (mouse; Sigma MABE1798; 1:500) - Validated by positive signal in cells known to express it and no signal in knockout cells<br>β-catenin (mouse; BD 610154; 1:50) - Validated by positive signal in cells known to express it<br>E-cadherin (rabbit; CST 3195, 1:200) - Validated by positive signal in cells known to express it<br>CDX2 (mouse; Atlas AMAb 91828, 1:1000) - Validated on manufacturers website by the provider<br>EpCAM-APC (BioLegend 118213; 1:200) - Validated on manufacturers website for FACS analysis of mouse tissue<br>LY6D-PE (BioLegend 138603; 1:200) - Validated on manufacturers website for FACS analysis of mouse tissue<br>LY6D-APC (Miltenyi 130-115-313; 1:50) - Validated on manufacturers website for FACS analysis of mouse tissue<br>ITGA5-PE (BioLegend 103805; 1:200) - Validated on manufacturers website for FACS analysis of mouse tissue<br>EpCAM-APC (BioLegend 324207; 1:50) - Validated on manufacturers website for FACS analysis of human tissue<br>LY6D-FITC (Cusabio Biotech CSB-PA613492LC01HU; 1:50) - Validated on manufacturers website for FACS analysis of human tissue<br>anti-rabbit-594 (Invitrogen A21207; 1:400) - Validated on manufacturers website by the provider<br>anti-chicken-488 (Invitrogen A78948; 1:400) - Validated on manufacturers website by the provider<br>anti-streptavidin-647 (Invitrogen S32357; 1:400) - Validated on manufacturers website by the provider<br>anti-rabbit-488 (Abcam 150073; 1:400) - Validated on manufacturers website by the provider<br>anti-b-actin (CST 4970; 1:5000) - Validated on manufacturers website by the provider<br>anti-rabbit IgG HRP-linked, (CST 7074; 1:5000) - Validated on manufacturers website by the provider<br>anti-mouseIgG HRP-linked, (CST 7076; 1:5000) - Validated on manufacturers website by the provider<br>anti-histone H3, acetyl K27 (rabbit; Abcam 1/250) - Validated on manufacturers website by the provider

Eukaryotic cell lines

Policy information about cell lines and Sex and Gender in Research

Cell line source(s)	AKP organoid line a gift from Jatin Roper and Omar Yilmaz lab. HEK293 cell line was used exclusively for the lentivirus production (see method section) and was kindly provided by Dr Juan Carlos Acosta (IGMM, Edinburgh), originally obtained from ATCC. BPN organoid line was a gift from Prof Owen Sansom.
Authentication	AKP line was authenticated by confirming mutational status of Apc (growth in absence of Wnt ligand), Kras (growth in absence of EGF) and P53 (growth in presence of nutlin). HEK293 cells were not authenticated independently.
Mycoplasma contamination	Cell lines and organoid cultures were routinely tested for mycoplasma contamination and found to be negative.
Commonly misidentified lines<br>(See ICLAC register)	Not used.

Animals and other research organisms

Policy information about studies involving animals; ARRIVE guidelines recommended for reporting animal research, and Sex and Gender in Research

Laboratory animals	Mice were purchased from Charles River and maintained at the animal facilities of the University of Edinburgh or Scotland Cancer Institute and were kept in 12 h light–dark cycles and were given access to water and food ad libitum. Mice were maintained in a temperature- (20–26 °C) and humidity- (30–70%) controlled environment. Mice were either C57Bl6J or CD1 nude. Female mice were used for all experiments at an age of between 6 and 12 weeks once they had reached a minimum weight of 20 g. At experiment endpoints (15mm for subcutaneous injection) mice were humanely sacrificed by cervical dislocation in line with UK Home Office regulations.
Wild animals	Not used in this study.
Reporting on sex	Female mice were used in this study as the AKP organoid line is derived from a female mouse thus enabling transplantation into immune competent donors.
Field-collected samples	Not used in this study.
Ethics oversight	All animal experiments were performed in accordance with a UK Home Office licences (PP9016178, PP7510272, PP3908577), and were subject to review by the animal welfare and ethics board of the University of Edinburgh and University of Glasgow.

Note that full information on the approval of the study protocol must also be provided in the manuscript.

Plants

Seed stocks	Not used in this study.
Novel plant genotypes	Not used in this study.
Authentication	Not used in this study.

Flow Cytometry

Plots

Confirm that:

☒ The axis labels state the marker and fluorochrome used (e.g. CD4-FITC).

☒ The axis scales are clearly visible. Include numbers along axes only for bottom left plot of group (a 'group' is an analysis of identical markers).

☒ All plots are contour plots with outliers or pseudocolor plots.

☒ A numerical value for number of cells or percentage (with statistics) is provided.

Methodology

Sample preparation	Pelleted organoids were resuspended in 1 ml TrypLE Express (GIBCO) and incubated at 37°C for 15 minutes. Cells were vigorously dissociated via pipetting, resuspended in 10 mL advanced DMEM/F12, passed through a 40µm cell strainer and centrifuged at 300g for 5 minutes at 4°C. Single cells were washed with 0.1% BSA in PBS and stained with the following antibodies: EpCAM-APC (BioLegend 118213; 1:200), LY6D-PE (BioLegend 138603; 1:200) or LY6D-APC (Miltenyi 130-115-313; 1:50), ITGA5-PE (BioLegend 103805; 1:200).<br>Human samples:<br>Normal colorectal mucosa and tumour were sampled from freshly resected surgical specimens from patients diagnosed with colorectal cancer. Tissues were cut into small pieces and then incubated in Advanced DMEM/F12 supplemented with 1mg/ml collagenase type IV (Sigma), 0.5mg/ml, hyaluronidase (Sigma) and 10µM Y-27632 (Tocris) at 37C with vigorous shaking until the tissue was completely disaggregated (60–90 min). The digested reaction was then filtered through a 70µm cell strainer. The filtered cells were centrifuged at 500g for 5 minutes, washed twice in Advanced DMEM/F12 and once in 0.1% BSA in PBS. Single cell suspension was then analysed by FACS using the following antibody: EpCAM-APC (BioLegend 324207; 1:50) LY6D-FITC (Cusabio Biotech CSB-PA613492LC01HU; 1:50).<br><br>Single viable cells were gated based on FSC and SSC/Single Cells (FSC-A/FSC-H)/Living Cells (DAPI negative).
Instrument	BD FACSARIA II/BD LSR-Fortessa X-20
Software	FlowJo v10.8
Cell population abundance	Organoid samples were pure epithelial and cell population abundance (different lineages) ranged from 2-50%. In primary tumour samples, epithelial cell abundance was variable but in all cases sufficient to determine the abundance of different lineages.
Gating strategy	We gated cells based on FSC and SSC/Single Cells (FSC-A/FSC-H)/Living Cells (DAPI negative). Then gated on fluorescent markers, for example EPCAM, LY6D, ITGA5. Positive cells were gated by comparing to samples stained with fluorophore conjugated IgG to determine the negative stained population.

☒ Tick this box to confirm that a figure exemplifying the gating strategy is provided in the Supplementary Information.
