# Supplementary material for: Loss of colonic fidelity enables multilineage plasticity and metastasis
Source: Nature. 2025 Jun 4;644(8076):547–56. doi: 10.1038/s41586-025-09125-5 (PMC12350155; doi:10.1038/s41586-025-09125-5)
Supplement: Supplementary file 4 — Supplementary Tables 1–11. [file 41586_2025_9125_MOESM4_ESM.zip › 2023-12-22276B-s4/Supplementary Table legends.docx]

**Supplementary Tables**

**Table S1. AKP + TGFB vs AKP ATRX + TGFB RNAseq.**

RNAseq data of *AKP* vs *AKP Atrx^KO^* organoids following TGF-beta treatment.

**Table S2. AKP vs AKP ATRX RNAseq.**

RNAseq data of untreated *AKP* vs *AKP Atrx^KO^* organoids.

**Table S3. TissueEnrich signatures.**

Results of TissueEnrich analysis of *AKP* vs *AKP Atrx^KO^* organoid RNAseq. Listed genes are those identified as markers of tissue listed. TissueEnrich analysis of scRNAseq clusters 4 (oesophagus) and 15 (skin) also shown.

**Table S4. AKP vs AKP ATRX scRNAseq cluster markers.**

Genes identifying scRNAseq clusters shown.

**Table S5. scRNAseq signatures.**

Gene signatures used for scRNAseq analysis listed.

**Table S6. ATACseq FDR0.01 results.**

Results of *AKP* vs *AKP Atrx^KO^* ATAC-seq. Note: control samples denote *AKP Atrx^KO^* samples, treatment samples denote *AKP* samples.

**Table S7. H3K27ac_diffbind_all_sites.**

Results of *AKP* vs *AKP Atrx^KO^* H3K27sc CUT&RUN analysis.

**Table S8. AKP vs AKP HNF4A RNAseq.**

RNAseq data of *AKP* vs *AKP Hnf4a^KO^* organoids

**Table S9. Clinical characteristics.**

Clinical characteristics of patient samples used for EPCAM/LY6D flow cytometry analysis.

**Table S10. HOMER TF enrichment human ATAC.**

HOMER transcription factor motif enrichment in *Atrx^KO^* signature high ATAC-seq analysis.

**Table S11. Primer sequences.**

Primer sequences used in this study.
